# TREATMENT OF SCAPHOID NONUNION WITH OLECRANON BONE GRAFT AND COMPRESSION SCREW

**DOI:** 10.1590/1413-785220162403155935

**Published:** 2016

**Authors:** ANTONIO TUFI NEDER, EDUARDO TRALDI FRANCESCHINI, ARLINDO GOMES PARDINI, MARCELO RIBERTO, NILTON MAZZER

**Affiliations:** 1. Hospital Lifecenter, Belo Horizonte, MG, Brazil.; 2. Hospital Ortopédico, Belo Horizonte, MG, Brazil.; 3. Universidade de São Paulo, Faculdade de Medicina de Ribeirão Preto. Ribeirão Preto, SP, Brazil.

**Keywords:** Scaphoid bone, Pseudarthrosis, Bone transplantation.

## Abstract

**Objective:**

To evaluate the outcome of olecranon bone graft and compression screw for the treatment of nonunion of the Lichtman type I scaphoid.

**Method:**

We evaluated 15 patients of 32 who underwent surgical treatment for nonunion of the Lichtman type I scaphoid with olecranon bone graft and screw compression.

**Results:**

We obtained 100% consolidation in our sample. The mean flexion of the wrist on the affected side was 68° and 75° on the non-affected side. The average extension was 63° and 72°, respectively. The average grip strength was 35 kgf. This corresponds to 98% of the handgrip strength of the non-affected side, which was 37 kgf. The DASH score averaged 5 points.

**Conclusion:**

We believe that the use of bone graft obtained from the olecranon and secured with cannulated screw is a resolute technique for cases of linear nonunion of the Lichtmann type I scaphoid. It has the advantages of a new anesthesia for removal of the graft and the access is easy, providing a good exposure for removal and good aesthetic results. ***Level of evidence IV. Case series.***

## INTRODUCTION

The scaphoid bone is the most commonly fractured carpal bone, corresponding to 60 to 70%^1^ of these fractures. It ranks second in relation to the wrist bones, radial distal fracture^1^ being the most common. This fracture often goes unnoticed in the acute phase due to various factors including stress and the difficulty to be evaluated by plain radiography. Moreover, the doctor's tendency is to concern about the most frequent fracture, which is the distal radial fracture.^2^ Heo et al.^3^ reported an incidence of 20.9% of fractures of the carpal bones associated to distal radial fractures.

Approximately 80% of the scaphoid bone surface is covered with cartilage. The main arterial blood supply is retrograde coming from the radial artery branch. Therefore, in the scaphoid fracture, the arterial blood supply to the proximal pole may be interrupted.^2^

The difficult diagnosis of scaphoid fracture can often lead to consolidation by intramembranous ossification. Immobilization is necessary for the proper formation of bone callus. The complications are associated with forces that cause this bone to move that, associated with its delicate vasculature, causing a high rate of nonunion.^1^ If undiagnosed or not treated properly, the natural evolution tends to nonunion, with subsequent posterior arthrosis and instability, leading to carpal collapse, known as "scaphoid nonunion advanced collapse" (SNAC). It has a high risk of necrosis of the proximal pole due to reduced vascularity.^4^

The gold standard treatment of nonunion of the scaphoid without arthrosis or instabilities, classified as Lichtmann grade I, is still the Matti-Russe technique, which uses cancellous bone graft to help consolidation.^5^

In this study we assessed patients with Lichtmann grade I scaphoid nonunion undergoing volar treatment with cancellous bone graft taken from the olecranon and fixed with cannulated screw.

## MATERIALS AND METHODS

In this study 52 patients undergoing surgery for scaphoid nonunion were evaluated from the hospital's database from January 2008 to December 2012. Inclusion criteria selected patients with Lichtmann grade I nonunion. Exclusion criteria were other levels of the aforementioned classification, patients with fractures in the proximal pole, previous wrist injury, instabilities, trans-scaphoid perilunate fracture dislocation, and necrosis of the proximal pole. Of the 52 initial patients 32 were selected, and only 15 showed up for evaluation. Thirteen patients have not been found and four refused to participate since they had no complaints. The selected patients were surgically treated in our hospital between January 2008 and December 2013. Therefore, these patients were at six months to five years postoperative period. All of them were surgically treated by volar fixation with cannulated compression screws and olecranon cancellous bone graft.

**Figure 1:**
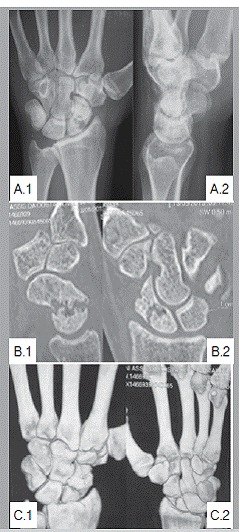
(A1 and A2) Preoperative anteroposterior and profile x-ray of the wrist (patient A) showing scaphoid nonunion, with no signs of arthrosis or Lichtmann grade 1 carpal instability; (B1 and B2) Sagittal and coronal sections Computed Tomography with good delimitation of bone resorption of nonunion focus without major angular change. (C1 and C2) 3D Reconstruction images of the wrist.

The study was approved by the hospital ethics committee, protocol CAAE 47957015.0.0000.5126, and all patients signed a Free and Informed Consent form before the surgery. Data on age, affected side, gender, and DASH test results were recorded. These 21 patients were tested for their ability to perform specific functions and checking for symptoms. They were tested for mobility by flexion and extension range of motion both for the affected and the contralateral wrist using a goniometer. Pain resulting from fossa radialis (anatomical snuffbox) palpation or thumb pistoning was also recorded. The wrist prehension strength tests compared to the contralateral side were performed using a Jamar dynamometer (Sammons Preston, Bolingbrook, IL, USA). These tests were performed three times on each side with the elbow flexed 90° and forearm in neutral rotation. The results were divided by three to obtain the final average for each side.^6^ The radiographic evaluation was made through the images in the following positions: neutral PA; PA with ulnar deviation of the wrist; profile; semi pronation oblique; and semi-supination oblique. (Figure 1) 

**Figure 2:**
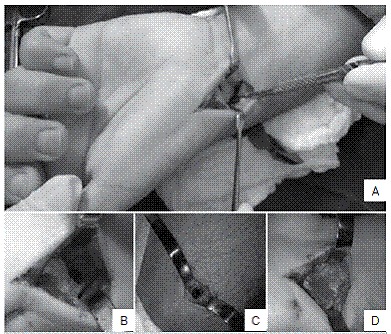
Intra-operatory photos (Patient A ). A: Volar access between carpal radial flexor and radial artery and aspect of bone resorption at scaphoid nonunion; B: Curetted nonunion focus; C: Elbow access for removal of olecranon bone graft; (D) Scaphoid nonunion focus filled with graft.

The fracture was considered consolidated according to the presence of trabecular bone in at least three views without pain on the fracture site.^7^ We considered DISI when the scapholunate angle was larger than 60° or the radio-lunar angle was larger than 30°.^8^ Surgery was performed with the patient in the supine position under brachial plexus anesthesia associated with sedation and pneumatic tourniquet. The volar access was between the flexor carpi radialis and the radial artery,^9^ exposing the nonunion focus, local curetted, fixated with a wire guide in the longitudinal axis of the scaphoid with fluoroscopic confirmation and another anti rotational wire parallel to the first one. We indirectly confirmed the screw size with a second guidewire. A tunnel was drilled with a drill and a circa 2cm longitudinal dorsal access was done in the proximal third of the forearm over the ulna, 4 cm distal to the olecranon apex and a circa 1cm² window was opened with an osteotome in the dorsal cortical bone of the proximal ulna and a curette graft was inserted for collection of cancellous bone. Impacted bone graft in the focus of the scaphoid nonunion and a screw was inserted with intra-focal compression. (Figure 2)

**Figure 3:**
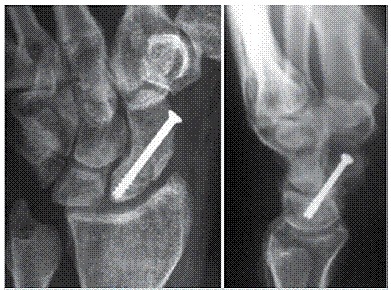
Antero-posterior and profile x-ray of wrist at one month postoperative with no signs of graft reabsorption. Patient A .

The position and size of a suitable screw were confirmed under fluoroscopy. Suture was done with nylon 4-0. Dressing with open gauze and placed plaster cast covering proximal phalanx from the thumb to the forearm. After 15 days the stitches were removed, but the plaster cast was maintained for six weeks. After six weeks and confirming the consolidation through radiographs, the plaster cast was removed and physiotherapy sessions for range of motion gain were started. As in preoperative evaluation, (Figure 1) intra-operative images (Figure 2) and one month postoperative images were taken (Figure 3).

## RESULTS

Of the 15 operated patients who attended evaluation consultation, all were men aged 20-38 years (mean 26 years old), the affected side was the right side in eight patients and the left side in seven patients. The mean follow-up time ranged between six months and five years between January 2008 and January 2013.

Of the revised medical records, both clinical and radiographic consolidation occurred at all patients without surgical re-intervention. No infections, dehiscence or pain complaints at the graft removal site have been reported. In the final clinical evaluation no functional disability, signs of scaphoid necrosis or posttraumatic arthrosis and thumb stiffness were reported. All patients informed that they continue to perform their prior to surgery activities.

As post-surgical complication we report one patient, at the seventh postoperative month, who showed marked loss of flexion-extension of the wrist, between 31° extension and 28° flexion, probably due to not having followed postoperative rehabilitation guidelines.

In the range of motion assessment, the mean wrist flexion at the affected side was 68° and the non-affected side 75° (range 49° to 75°). The mean extension was 63° on the affected side and 72° of the non-affected side (ranging from 47° to 69°). The mean grip strength was 35 kgf, corresponding to 98% of the non-affected hand (37 kgf, ranging from 33 kgf to 38 kgf). (Table 1)

**Table 1:** Results.

Patient	Prehension strength Affected side/Non-affected side (Kgf)	Pain at anatomical snuffbox palpation or thumb pistoning	Arc of movement Flexion (degrees)	Extension Affected side/Non-affected side	DASH (points)
1	34/35	No	73/75	66/73	0
2	37/36	No	68/74	64/71	3
3	35/37	No	75/76	67/74	0
4	34/36	No	70/77	64/75	10
5	34/40	No	49/80	47/75	18
6	34/37	No	71/73	66/70	7
7	35/35	No	66/74	60/71	0
8	35/39	No	65/73	60/70	8
9	36/38	No	68/76	63/72	2
10	34/37	No	67/74	64/69	15
11	38/34	No	70/72	65/69	0
12	35/36	No	68/76	64/73	0
13	35/40	No	67/74	62/72	8
14	37/37	No	72/75	69/72	0
15	32/38	No	71/76	64/74	4

The DASH functional test showed average score of 5 points. To one patient, whose leisure activity was playing the trumpet and conducting an orchestra, the specific questionnaire for musicians was applied. He scored zero at both the specific and non-specific questionnaires. (Table 1)

Table 2 shows the descriptive analysis of grip strength, range of flexion-extension and DASH questionnaire results. We found that only DASH showed high variability, because the variability coefficient is greater than 50%. The average DASH score was 5.0 points (ranging from 2.0 to 8.0). 

**Table 2:** Complete descriptive for prehension strength, arc of movement, extension and DASH.

Descriptive	Prehension strength		Arc of movement		Extension		DASH
Affected side	Non-affected side	Affected side	Non-affected side	Affected side	Non-affected side
Mean	35.0	37.0	68.0	75.0	63.0	72.0	5.0
Median	35	37	68	75	64	72	3
Standard deviation	1.5	1.8	5.9	2.0	5.0	2.0	5.9
CV	4%	5%	9%	3%	8%	3%	117%
Min	32	34	49	72	47	69	0
Max	38	40	75	80	69	75	18
N	15	15	15	15	15	15	15
CI	0.8	0.9	3.0	1.0	2.5	1.0	3.0

We used the Wilcoxon test to compare the results on strength, arc of flexion and extension of the wrist between affected and unaffected sides. This test was used because the data is paired, i.e., when the same subject is research and control of himself; *p* -value is the result of each comparison. (Table 3)

**Table 3:** Side comparison for strength arc of movement, flexion and extension.

Side		Mean	Median	Standard deviation	N	CI	p-value
Prehension strength	Affected side	35.0	35	1.5	15	0.8	0.017
	Non-affected side	37.0	37	1.8	15	0.9	
Flexion	Affected side	68.0	68	5.9	15	3.0	0.001
	Non-affected side	75.0	75	2.0	15	1.0	
Extension	Affected side	63.0	64	5.0	15	2.5	0.001
Non-affected side	72.0	72	2.0	15	1.0

## DISCUSSION

Scaphoid fractures often go unnoticed through the initial X-rays, that detect only about 70% of them. Other diagnostic tests include computed tomography, bone scintigraphy, MRI, and ultrasound.^2^ MRI was considered the gold standard^5^ and computed tomography with technological development has gained more diagnostic importance.^10^ Yildirim et al.^11^ suggested that ultrasound evaluation in the emergency room would increase the diagnostic power of this injury.

The fracture mechanism was generally fall with the flat hand in dorsiflexion and radial deviation of the wrist.^12^ There are a number of treatment techniques for scaphoid nonunion without signs of SNAC or necrosis of the proximal pole (Lichtmann grade I), such as percutaneous screws fixation with or without bone graft,^10^ fixation with Kirschner wires,^13^ vascularized bone graft,^9^ ^,^ ^14^ ^,^ ^15^ and others that report using different types of screws.

Despite advances in surgery and in the understanding of bone healing, bone nonunion remains a challenge. Autologous bone grafts is still a standard with low postoperative pain in the donor area, good aesthetic result with a 1.5-2.0 cm longitudinal scar at the posterior elbow region. Moreover, it is a ​​little exposed area for graft extraction reducing, thus, the risks of another anesthesia. It is a surgically accessible region with good exposure for the small amount of soft tissue. The consolidation ratio in this study was 100% within the standards for autologous bone graft.^16^

We believe that a suitable patient selection for this type of surgery is important. We do not use this technique in patients with fractures of the proximal pole, tuberosity fractures, previous wrist injury, scaphoid semilunate dissociation, scaphoid perilunate fracture dislocation, humpback deformity, or avascular necrosis. It was important, at the radiographic evaluation, an angle measurement: if the radio-lunate angle is greater than 30° and the radius scapholunate is greater than 60°^10^ it was necessary to employ the Fisk-Fernandez technique to correct DISI^5^ using a wedge graft bone from the iliac crest. For advanced degrees of SNAC or necrosis of the proximal pole other techniques may be indicated, such as rescue techniques, including the four corners arthrodesis,^4^ resection of the first carpal row, radiocarpal arthrodesis,^17^ pure scaphoid excision^18^ or the pyrocarbon prosthesis,^4^ ^,^ ^17^ ^,^ ^19^ considering the proper indication in each case.

In our series we chose to use bone graft taken from the olecranon because we considered that this option dispenses the approach in other body segments, limiting the procedure to the limb to be operated, thus, avoiding additional anesthesia. Moreover, it is a donor region easily accessible with little visual exposure, favoring the aesthetic aspect.

The limitations identified in this study were the small number of cases due to the fact that scaphoid nonunion is relatively rare, allied to the difficulty in getting patients to return for evaluation.

In the postoperative radiographic evaluation no patients presented DISI or signs of SNAC and arthrosis. All patients showed signs of trabecular bone in at least three radiographic views. Signs of impact in the trapezium screw head were found in four patients with bone remodeling at the trapezoid base, but none complained or had functional restrictions. The scaphoid nonunion was due to lack of stability and adequate reduction, to a vascularity reduction and also the forces the wrist bone is subject. ^20^ The delay in proper immobilization for more than one month in the case of acute fracture would increase this incidence.^12^ Compression screws to the scaphoid in cases of nonunion showed excellent results with 91-100% consolidation rates.^10^ ^,^ ^13^

Abassi et al.^21^ enumerated the advantages of screw fixation, including acute fractures, with accelerated rehabilitation and early return to work and good results. Ya'ish et al.^17^ described advantages in carrying out this fixation with bioabsorbable screws, as reduced risk of later removal of the implant, thus, avoiding the difficulties and risks of a surgery. According to Kirkham and Millar^13^ fixation with compression screws in cases of DISI and cortical-cancellous bone graft is technically demanding. According to their research results with fixation only with Kirschner wires are compatible or even better as compared to other techniques. Léon et al.^14^, Malizos et al.^22^ and Liang et al.^23^ showed 100% consolidation with vascularized bone graft based on different arterial supply with this type of treatment, being more indicated necrosis of the proximal pole associated with nonunion as an attempt to improve bone blood supply.

Regarding DASH score, mean value was 5 ± 5 points which is considered an excellent end result, since the lower the score, the lower the degree of dysfunction. Kim et al. presented in their study DASH score 9 ± 6 using only volar percutaneous screw for treatment of scaphoid nonunion.^10^

**Figure 4:**
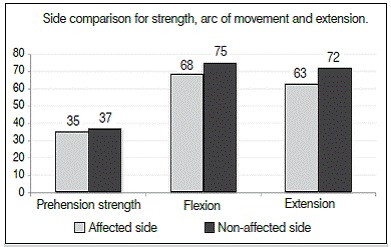
Side comparison for strength, arc of movement and extension.

The dynamometer tests showed a mean of 35 kgf, corresponding to 98% of unaffected hand strength, 37kgf (Figure 4), which remained within the normal range according to the literature.^6^

The average decrease in bending the affected side compared to the normal side was 7° (affected side 68° and unaffected side 75°) and the average decrease of the extension was 9 (affected side 63 and side affected not 72° ) which was considered a good result (Figure 4). This was also confirmed by the low DASH score of patients.

The only patient who presented considerable loss of wrist range of motion was on the seventh postoperative month and had not followed the guidelines of medical rehabilitation. He showed loss of flexion-extension accented handle (31° extension and 28° flexion) compared to the normal side, thus demonstrating the need for proper rehabilitation after surgery.

## CONCLUSION

The small incision, lower visual exposure, and scar location on the back of the elbow are important aesthetic factors. Bone healing with preservation of limb functionality was obtained in all cases. The use of olecranon bone graft and cannulated screw fixation was a successful technique for Lichtmann type I scaphoid nonunion. We obtained a statistically significant difference between affected and non-affected sides regarding strength, arc of flexion and extension.
